# A Randomized Controlled Trial of Changes in Fluid Distribution across Menstrual Phases with Creatine Supplementation

**DOI:** 10.3390/nu15020429

**Published:** 2023-01-13

**Authors:** Sam R. Moore, Amanda N. Gordon, Hannah E. Cabre, Anthony C. Hackney, Abbie E. Smith-Ryan

**Affiliations:** 1 Applied Physiology Laboratory, Department of Exercise and Sport Science, University of North Carolina at Chapel Hill, Chapel Hill, NC 27599, USA; 2 Human Movement Science Curriculum, Department of Health Sciences, University of North Carolina at Chapel Hill, Chapel Hill, NC 27599, USA; 3 Department of Nutrition, Gillings School of Global Public Health, University of North Carolina at Chapel Hill, Chapel Hill, NC 27599, USA

**Keywords:** dietary supplement, female physiology, total body water

## Abstract

This study examined the effects of creatine (Cr) loading on body mass (BM) and fluid markers of total body water (TBW), extra-cellular fluid (ECF), and intra-cellular fluid (ICF) across the menstrual cycle (MC). Thirty moderately active females, either naturally-menstruating (NM) or using hormonal contraceptives (HC), were randomized to Cr (Cr; 4 × 5 g/day of creatine monohydrate for 5 days; *n* = 15) or a non-caloric placebo (PL; *n* = 15) using a double-blind, placebo-controlled design, with a menstrual phase crossover. BM, TBW, ECF, and ICF were measured at pre- and post-supplementation in randomized order of follicular phase (FP; NM: MC days 0–8, HC: inactive pill days) or luteal phase (LP; NM: ≤15 days from next projected cycle start date, HC: active pill days) using bioelectrical impedance spectroscopy. Acute hydration status and salivary estrogen were used as covariates. Change in BM was not different between groups across MC ([PL-Cr] Δ 0.40 ± 0.50 kg; *p* = 0.427) or between MC phase across groups ([FP-LP] Δ 0.31 ± 0.48 kg; *p* = 0.528). TBW (*p* = 0.802), ECF (*p* = 0.373), and ICF (*p* = 0.795) were not different between supplement groups at pre-supplementation/FP time points. There were no significant differences between the NM and HC subjects at any time point, for any outcome (*p* > 0.05). Following LP supplementation, significant changes were observed in TBW (Cr: Δ 0.83 ± 0.38 L, PL: Δ −0.62 ± 0.38 L; *p* = 0.021), ECF (Cr: Δ 0.46 ± 0.15 L, PL: Δ −0.19 ± 0.15 L; *p* = 0.013), and ICF (Cr: Δ 0.74 ± 0.23 L, PL: Δ −0.02 ± 0.23 L; *p* = 0.041). These data demonstrate an increase in all fluid compartments in the LP following Cr loading, without observed alterations in body weight for females.

## 1. Introduction

Despite women being the largest group of dietary supplement consumers in the United States, with more than 77% of women reporting supplement use, the representation of women in supplement ingredient research is far from equitable [1]. Creatine monohydrate (Cr) is one of the most widely studied dietary supplements with over 500 peer-reviewed publications [2], and has gained recent attention for providing unique benefits to females [3]. Despite the growing evidence of benefits, a recent survey showed less than 20% of females used Cr [4]. Women are often hesitant to use creatine in part due to a fear of weight gain, an unsubstantiated side effect in biological females, primarily observed in male populations [5]. While research in females with Cr supplementation is growing [3], the effects of Cr across the menstrual cycle (MC) are currently unknown.

Cr supplementation provides an array of physiological benefits, with exercise performance-related benefits more highly touted [5], specifically related to delayed fatigue during repeated high-intensity exercise [6]. Other benefits of Cr use in female populations include a greater efficacy to anti-depressants [7], augmented cognitive function [8], and improved working memory in non-meat eaters [9], all outcomes that can fluctuate throughout the MC [10,11]. Despite a widespread consensus on safety in the literature, many misconceptions and myths regarding Cr still exist [2]. Most commonly, the myth is that Cr supplementation leads to an increased instance of dehydration and muscle cramping [12]. However, evidence demonstrates a 60% decrease in muscle cramps during hemodialysis with Cr supplementation during a double-blind placebo-controlled study in male and female subjects with end-stage renal disease [13]. This is likely due to the molecular structure of Cr, which can be characterized as an osmotically active substance, effectively “pulling” water into the intracellular space [14] exhibited by significant increases in intracellular fluid (ICF) and total body water (TBW) volumes in male subjects [15]. The fluid shifts that occur with Cr supplementation have also positively impacted thermoregulation and performance in hot and humid conditions [12]; however, these outcomes have only been explored in male populations.

Estrogen and progesterone directly impact fluid distribution across the MC, demonstrating a greater extracellular shift when hormones are elevated, particularly during the luteal phase (LP) [15]. Changes in fluid markers in the LP may negatively influence thermoregulation and exercise performance [16]. Cr supplementation has previously been shown to increase TBW by pulling water into the cell [17], which may be beneficial to counteract the extracellular shift reported during the LP. However, the impact of Cr on fluid distribution in the LP has yet to be explored in females. One previous study evaluated a mixed sample of males and females and reported a significant increase in TBW and body mass (BM), with no effect on ICF, after a 7-day loading, and 21-day maintenance supplementation protocol, only controlling for the MC phase at the pre-supplementation time point [18]. While prior mixed-sex studies demonstrated improved hydration-related outcomes with Cr loading in the follicular phase (FP) [19], the shift in fluid distribution that occurs in the LP highlights the need for a further understanding of how Cr supplementation may impact fluid distribution in females across the MC. To our knowledge, the influence of Cr on fluid distribution across the MC has not yet been evaluated, which could have important implications for active women. Therefore, the purpose of this study was to examine the effects of Cr loading on fluid balance through markers of TBW, ECF, and ICF across MC phases. Based on previous research, we hypothesized increased levels of fluid markers (TBW, ECF, and ICF) as a result of a Cr loading period, with the potential for a greater magnitude of change in the LP.

## 2. Materials and Methods

### 2.1. Subjects

**Figure 1:**
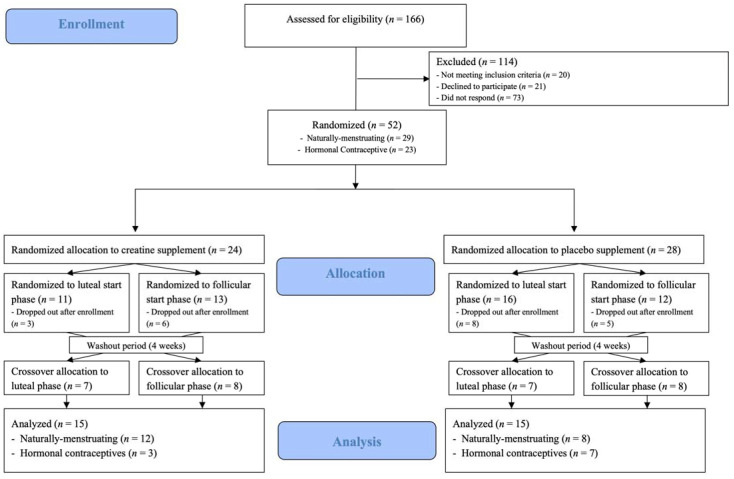
CONSORT (Consolidated Standards of Reporting Trials) diagram.

Of the 43 moderately active (self-reported a minimum of 30 min per day for 3 or more days per week of moderate to vigorous exercise, determined from the PAR-Q) female subjects that were enrolled, 30 subjects (mean ± standard deviation [SD]), Age: 25 ± 6 yrs; Height: 165.8 ± 5.4 cm; BM: 64.5 ± 10.1 kg; BMI: 23.4 ± 2.4 kg/m^2^) completed this randomized, double-blind, placebo controlled study, with a menstrual phase crossover component (Figure 1). Inclusion criteria for participation included: BMI between 18.5 and 29.9 kg/m^2^, 18–40 years of age, participating in moderate to vigorous intensity exercise at least 3 days per week, generally healthy as identified by a health history questionnaire, and either naturally menstruating (NM; [*n* = 20] or using non-hormonal copper intrauterine device [*n* = 1]), or using a hormonal contraceptive (HC: monophasic combination oral contraceptive [*n* = 6] or hormonal intrauterine device [*n* = 3]; Table 1). Inclusion requirements for NM subjects included that they experienced regular menstrual cycles between 21 and 35 days in length, for at least 3 months prior to the beginning and throughout the duration of the research study. Subjects were excluded if they were amenorrheic (lack of menstruation for ≥3 consecutive cycles) or oligomenorrheic (MC length ≤36 days) [20] as determined by menstrual cycle tracking and/or health history questionnaire; experienced a musculoskeletal injury in the three months prior to the study; were currently or trying to become pregnant; unwilling to abstain from taking NSAIDs or consuming >200 mg of caffeine per day during the study; or using performance-enhancing supplements (creatine, protein, beta-alanine, carnosine, taurine, etc.). Reasons for attrition included: change in hormonal contraceptive type (*n* = 3); decreased schedule availability (*n* = 3); onset of illness or musculoskeletal injury (*n* = 3); positive pregnancy test (*n* = 1); and reasons undisclosed to the research team (*n* = 3). Both NM subjects and those using HC, with controlled timepoints, were enrolled in order to improve the generalizability to females. Sample size estimation was determined a priori on the primary outcome variables of fluid distribution with an assumed average effect size of 0.45 and a power of 0.80 [15,18,21,22,23], requiring a total sample size of 42 (*n* = 21 per treatment) based on previous studies. Post hoc power calculations demonstrated an average effect size of 1.28 and power of 0.90, demonstrating adequate power.

### 2.2. Experimental Design

**Figure 2:**
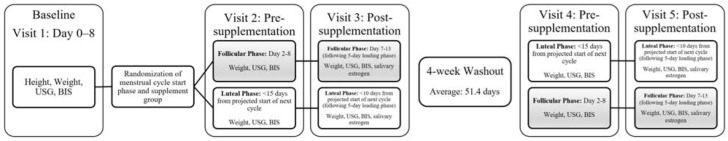
Experimental design [Urine Specific Gravity (USG), Bioelectrical Impedance Spectroscopy (BIS)]. Subjects were randomized to supplement group (PL or Cr) and hormonal phase order, with a minimum 4-week washout period to ensure testing across multiple cycles and account for familiarization. Note: Subjects were randomized to supplement group (PL or Cr) and hormonal phase order, with a minimum 4-week washout period to ensure testing across multiple cycles and account for familiarization.

Subjects were asked to arrive at the University of North Carolina at Chapel Hill Applied Physiology Laboratory following a 12-h fast from all caloric foods and beverages and a 48-h abstention from vigorous physical activity. Prior to arrival, the subjects were randomized, using a block randomization software (Sealed Envelope software, London, UK) to a Cr loading (4 × 5 g for 5 days) or non-caloric placebo (PL; 4 × 3.5 g Crystal Light) group; supplementation groups were maintained for the duration of the study. In a crossover approach, subjects were randomly assigned to MC, starting in either FP (days 0–8 of the cycle) or LP (within 15 days from the next projected cycle start date) (Figure 2). The initial supplementation period was followed by a minimum 4-week washout before the subjects returned for the supplementation period in the alternate menstrual phase. Subject height and weight were measured upon arrival at the laboratory via a portable stadiometer (Perspective Enterprises, Portage, MI, USA) and electronic scale (InBody 770, BioSpace, Seoul, Republic of Korea), respectively. Fluid measurements (TBW, ECF, and ICF) were measured using bioelectrical impedance spectroscopy (BIS). Hydration was measured immediately upon arrival and prior too BIS assessment through urine specific gravity using a refractometer to ensure that the subjects were in the euhydrated range (1.002–1.025); if subjects were dehydrated (>1.025), they were asked to consume 16 oz of water and re-test after a 15 min waiting period, and if subjects were hyperhydrated (<1.002), their visit was rescheduled. All procedures were approved by the University’s Institutional Review Board for Human Subjects (IRB #21-1423), and all subjects signed written informed consent prior to participation. This clinical trial was registered at Research for Me (Record ID: 3038-21-1423).

### 2.3. Bioelectrical Impedance Spectroscopy (BIS)

Fluid measurements of TBW, ECF, and ICF were obtained at each of the five visits using a multi-frequency BIS device (SFB7 ImpediMed, Queensland, Australia [10–500 kHz]). Subjects were asked to lay supine on the exam table with electrodes placed 5 cm apart at the right wrist and ankle, respectively. Height, weight, age, and sex were entered into the device prior to testing, during which the subject laid supine for 3–5 min prior to measurement to allow for the regulation of posture-related changes in fluid distribution. Device software reported TBW (L), ECF (L), and ICF (L), and an average of two trials was recorded. Reliability in our laboratory for these BIS outcomes within the current sample include: TBW: standard error of measure (SEM) = 0.708 L; ECF: SEM = 0.342 L; ICF: SEM = 0.454 L.

### 2.4. Hormonal Status

**Figure 3:**
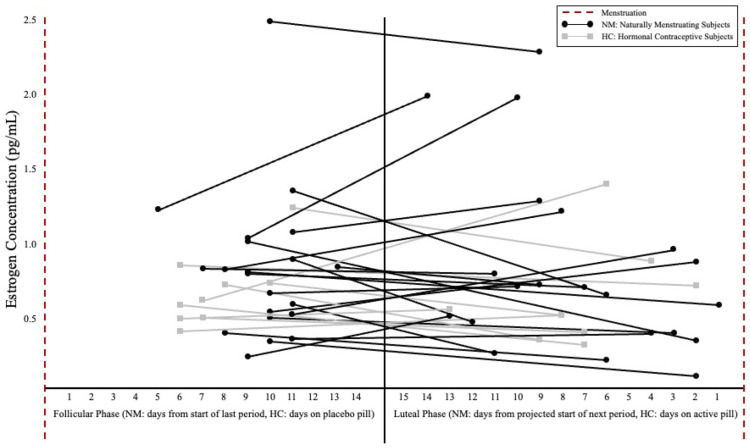
Estrogen concentration (pg/mL) and post-supplementation day of cycle between hormonal phases. Note: Follicular phase days were counted from the start of the subject’s last period or as days on a placebo pill for HC users. Luteal phase days were determined by number of days until the next projected period or days on the active pill for HC users. Black circles represent naturally menstruating subjects and gray boxes represent subjects using hormonal contraceptives. Connected dots indicate the spread of post-supplementation testing for individual subjects. There were no significant differences in estrogen concentration between NM and HC subjects or between hormonal phases (significance level set at ≤0.05).

For NM subjects, MC phase and length and ovulation date were determined using a menstrual cycle tracking app (FertilityFriend) to record the menses, symptoms, and basal body temperature, accessible by both the subject and research team. While ovulation kits were not used due to the specific time course of supplementation (Figure 2), estrogen concentration was collected to characterize the MC phase at each post-testing visit. Salivary estrogen levels were measured via 2.5 mL passive drool saliva collection and were analyzed by an ELISA assay (Salivary 17 β-Estradiol Enzyme Immunoassay Kit, Salimetrics, LLC, State College, PA, USA). The single subject using a non-hormonal copper intrauterine device was categorized as part of the NM group due to the presence of regular menses prior to, and over the duration of the study, aligning with prior research indicating no influence of copper intrauterine devices on serum estrogen or progesterone levels [24]. Subjects using HCs were evaluated during their inactive pill week for the FP and active pill week for the LP measurements. Individual variability for estrogen concentration and corresponding cycle day for both post-testing visits can be found in Figure 3.

### 2.5. Supplementation

Subjects were randomized in a crossover design to the Cr group (Creapure® creatine monohydrate AlzChem, Trostberg GmbH, Germany, GRAS Notice No. GRN 931; 5 g creatine monohydrate + 2 g non-caloric Crystal Light), or a non-caloric PL (3.5 g Crystal Light). The same dosing pattern was followed for both groups (4 × 5 g for 5 days; 20 g total per day) consumed with 4–6 ounces of water, at regular intervals each day. Supplementation periods were separated by a minimum 4-week washout. Supplements were blinded and packaged in 2 oz. food grade black portion cups with clear lids by an individual who was not directly involved with supplement distribution. Both treatments were identical in texture, color, and taste and were distributed at the beginning of each MC phase pre-supplementation visit. Supplementation logs and empty containers were collected at the end of each loading phase to monitor compliance (Cr: 94.0%; PL: 99.8%).

### 2.6. Statistical Analysis

One-way analyses of variance tests were used to evaluate the baseline differences between naturally menstruating subjects and those using hormonal contraception. Repeated measures analyses of variance tests were used to analyze the change in BM following supplementation (Cr and PL) and across menstrual phases (FP and LP). Separate univariate analysis of covariance tests were used to assess the difference between treatment groups (Cr and LP) pre- and post-supplementation for TBW, ECF, and ICF, in each MC phase (FP vs. LP), using hydration status (USG) and estrogen concentration as covariates. The significance level was set at ≤0.05. Analyses were completed using SPSS (version 27, IBM, Armonk, NY, USA).

## 3. Results

There were no significant differences between the NM and HC subjects for BM, TBW, ECF, or ICF, at the pre-supplementation visits in the FP (*p* = 0.074–0.809) or LP (*p* = 0.061–0.509). There was no significant phase × supplement interaction for BM (Table 2), with no main effect for phase (mean difference [MD; FP pre- to post-supplementation Δ—LP pre- to post-supplementation Δ] ± standard error [SE]: 0.31 ± 0.48 kg; *p* = 0.528), and no main effect for supplement (MD ± SE [PL-Cr]: 0.40 ± 0.50 kg; *p* = 0.427). There were no significant differences in the estrogen concentration between the NM and HC subjects in either the FP (*p* = 0.573) or the LP (*p* = 0.964) (Figure 3) as well as no significant differences between the MC phases (*p* = 0.761). There were no significant differences for hydration at the baseline visits in FP (*p* = 0.919) or LP (*p* = 0.439).

### 3.1. TBW

**Figure 4:**
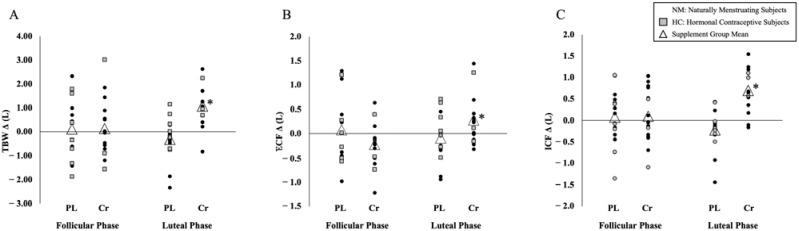
Individual pre- to post-supplementation change scores between hormone phases. (**A**) Total body water (TBW; L), (**B**) extra-cellular fluid (ECF; L), (**C**) intra-cellular fluid (ICF; L). Note: Change scores of individual responses and supplement group means of TBW, ECF, and ICF are shown. Black circles represent naturally menstruating subjects and gray boxes represent subjects using hormonal contraceptives. Triangles indicate the supplement group mean change score. * Significance (*p* ≤ 0.05) was noted from ANCOVA with change scores covaried for hydration (USG = 1.016) and estrogen concentration (0.791 pg/mL).

There was no significant difference between the MC phase for change in TBW (*p* = 0.769) prior to supplementation (MD ± SE [PL-Cr]: 0.226 ± 0.763 L). There was a significant difference for the change in TBW across the MC phase between the supplement groups (*p* = 0.021), with a greater increase in TBW in the LP for the Cr group (Δ 0.832 ± 0.376 L) compared to the PL group (Δ −0.616 ± 0.376 L). A significant group effect was also noted (*p* = 0.019) from pre- to post-supplementation in the LP for TBW with a significantly greater increase in the Cr group (Δ 1.168 ± 0.358 L) compared to PL group (Δ −0.231 ± 0.358 L; Figure 4A).

### 3.2. ECF

No significant difference was observed in the ECF change between the MC phase (*p* = 0.311) prior to supplementation (MD [PL-Cr] ± SE: 0.303 ± 0.294 L). There was a significant group effect for change in the ECF across the MC phase (*p* = 0.013), with a greater increase in ECF in the LP for the Cr group (Δ 0.460 ± 0.154 L) compared to the PL group (Δ −0.186 ± 0.154 L). A significant group effect was also observed (*p* = 0.015) from pre- to post-supplementation in the LP with a significant increase in the ECF in the Cr group (Δ 0.464 ± 0.155 L) compared to the PL group (Δ −0.171 ± 0.155 L; Figure 4B).

### 3.3. ICF

There was no significant difference between the MC phase for change in ICF (*p* = 0.809) prior to supplementation (MD [PL-Cr] ± SE: 0.124 ± 0.508 L). A significant difference for change in ICF across the MC phase was observed between the supplement groups (*p* = 0.041), with a greater increase in ICF in the LP for the Cr group (Δ 0.742 ± 0.227 L) compared to the PL group (Δ −0.024 ± 0.227 L). There was also a significant group effect (*p* = 0.039) for ICF with a greater increase in the Cr group (Δ 0.732 ± 0.231 L) over the PL group (Δ −0.054 ± 0.231 L; Figure 4C) in the LP supplementation phase.

## 4. Discussion

Creatine supplementation is often associated with suspected dehydration and muscle cramping; however, these are unfounded and have been primarily explored in men [12]. The efficacy of Cr has been attributed to cell swelling, primarily through increased ICF, due to the osmotically active structure of the Cr monohydrate [17] and increased TBW [23]. Increases in fluid markers with Cr supplementation are often accompanied by a significant and expected gain in BM, however, this outcome is predominantly observed in male populations [25]. To date, we are aware of no previous investigations exploring the effects of fluid distribution with Cr across the MC. Although some data have characterized fluid changes across the MC [26], the relationship and potential impact of variations in fluid distribution with Cr supplementation is not well-understood. The present study demonstrated significant increases in TBW (Δ 0.832 ± 0.376 L), ECF (Δ 0.460 ± 0.154 L), and ICF (Δ 0.742 ± 0.227 L) in the LP following Cr supplementation when compared to a PL control. These increased levels of fluid markers occurred with no significant changes in BM across the MC (MD ± SE: 0.31 ± 0.48 kg; *p* = 0.528) or following Cr supplementation (MD ± SE [PL-Cr]; 0.40 ± 0.50 kg; *p* = 0.666).

### 4.1. TBW

A commonly reported side effect of Cr supplementation is an increase in BM, ranging from 0.7 to 1.4 kg, in 7- and 5-day loading protocols, respectively [27,28]. The BM increases are often attributed to increased fluid retention, supported by significant increases in TBW, a beneficial parameter in muscular performance [29]. In a sample of 13 well-trained males, significant increases in TBW (2.3 L) and BM (1.1 kg) were reported following Cr supplementation, with no significant changes for the PL in either TBW or BM (2.3 L and 0.1 kg, respectively) [15]. These findings are consistent with one mixed-sex study that demonstrated a significant increase in BM (0.75 kg; *p* < 0.05) and TBW (1.37 L; *p* < 0.05) following a 7-day Cr loading phase when compared to the PL group [18]. Powers et al. (2003) found no significant sex interactions despite noting smaller fluctuations in TBW and BM in the female subjects, however, sex-specific data were not analyzed. Collectively, it appears that females do not demonstrate increases in BM following Cr supplementation; to our knowledge, the impact on TBW has not been directly studied in females. The present study resulted in no changes in BM following Cr supplementation when compared to PL (MD ± SE [PL-Cr]: Δ 0.40 ± 0.50 kg; *p* = 0.427) or between FP and LP menstrual cycle phases (*p* = 0.528). In contrast, significant increases of nearly a liter in TBW resulted following Cr supplementation in the LP (Δ 0.832 ± 0.376 L), demonstrating beneficial increases of hydration markers with no fluid-retention-related weight gain (Figure 4A).

### 4.2. ECF

Creatine monohydrate exhibits an osmotic effect that promotes intra-cellular swelling, favoring a shift to ICF under homeostatic conditions. Changes observed in TBW following Cr supplementation in males appear to systematically increase, while data characterizing the distribution of fluid between intra- and extra-cellular compartments are inconsistent [30]. While some short-term loading protocols result in no change in ECF (15,31), similar protocols have resulted in ECF increases of nearly half a liter (Δ 0.42 L) while reporting no change in fluid distribution [18]. Significant increases in ECF (Δ 1.18 L) were reported following a 5-day loading phase in endurance-trained males [21], while a 3-day protocol of 0.07 g Cr/kg FFM every three hours resulted in no significant increase in ECF (*p* = 0.51) [31]. The variation in duration and amount of Cr loading protocols has been hypothesized as a potential cause of ECF outcome discrepancies. The amount of time elapsed between the end of supplementation and when measurement occurs may be another possible explanation for differences in ECF outcomes. Ziegenfuss et al. (1998) conducted post-supplementation measurements within 24 h following the conclusion of the supplementation protocol and noted an insignificant change, while Weiss and Powers (2006) waited two days to conduct post-supplementation measures. The extended duration of time after supplementation may have impacted the fluid distribution, with increased intra-cellular concentrations resulting from Cr supplementation continuing to draw fluid intra-cellularly over the additional time. When evaluating the MC, research regarding fluid distribution provides inconsistent results within the MC phases [32,33,34]. There appears to be a consistent increase in ECF associated with increased levels of estrogen, seen around ovulation and LP. A 16.1% ECF increase was reported as a result of the suppressed transcapillary movement of albumin with the administration of estrogen, and a 17.2% increase in ECF with the combined administration of estrogen and progesterone when compared to a gonadotropin-releasing hormone antagonist [35]. These mechanisms underpin the outcomes of the present study, demonstrating a significant increase in ECF with Cr loading, particularly in the LP (Δ 0.464 ± 0.155 L; Figure 4B). The influx of ECF accumulation in the LP may be counteracted by the osmotic effect of Cr, which could be a potential mechanism for increased TBW despite no similar increase in BM for females using Cr in the LP.

### 4.3. ICF

The osmotic effect of the Cr monohydrate is well-documented in terms of ICF increases seen with Cr loading protocols. Nearly 60% of TBW is held intra-cellularly [36], citing a Cr-directed increase in osmotic pressure as the driver behind cellular swelling and ICF increases [17]. This is consistent with available studies in male only populations, with significant ICF increases seen in short-term Cr loading protocols ranging from 0.77 L (*p* < 0.001) [31] to ~1.60 L [15,21]. Powers et al. (2003) demonstrated a significant increase in ICF (0.95 L) after a 7-day loading protocol; female subjects were measured during the FP but were not characterized separately from the group analysis [18]. The impact of rising estrogen and progesterone levels in the LP resulted in decreases in the ICF volume [34]. The current study demonstrated a statistically significant increase in ICF at the LP post-supplementation time point compared to the FP post-supplementation time point (Δ 0.742 ± 0.227 L) and LP pre-supplementation time point (Δ 0.732 ± 0.231 L) in the Cr group (Figure 4C). These increases could represent an antagonistic action of creatine monohydrate pulling water into the cell, despite estrogen and progesterone influencing the distribution toward the extra-cellular compartment.

### 4.4. Limitations

No study is without limitations; the current study measured estrogen concentration at the post-supplementation time points only, which likely contributed to the lack of significance in salivary concentrations (Figure 3). Additionally, the inclusion of a progesterone measurement, which has also been shown to favor extracellular fluid retention [32], may have been helpful to further confirm the MC phase as well as provide further insights into the relationship between fluid distribution and female sex hormones. While there were no significant pre-supplementation differences between the NM and HC subjects, the authors acknowledge potential differences in the ovarian hormone profiles between the two groups. Ovulation kits were not utilized due to the intricacy of a 5-day supplementation period along with a 4-week washout. Despite not confirming ovulation, several other controls were in place to support MC phase estimation. Finally, the inclusion of both NM and HC subjects was intentional to improve the generalizability to a broader audience of females due to high rates of HC use. Based on the preliminary analyses across NM, HC, and IUD users, there appears to be no difference.

## 5. Conclusions

The present study demonstrates significant increases in TBW, ECF, and ICF as a result of Cr loading in the LP. These data demonstrate that Cr supplementation may improve hydration status in the LP, with no significant increase in BM, a common fear dissuading the use of Cr in female populations. A growing body of research demonstrates a significant impact of the MC on thermoregulation [26], resulting in increased cardiovascular strain, core temperature, and sweat rate all in the LP [37]. Prior research has demonstrated the relationship between increased TBW and ICF with improved exercise performance and thermoregulatory outcomes [17,29,38]. Though the current study did not explore the impact of fluid markers on thermoregulation, the improvements in fluid distribution reported may counteract the suppressed thermoregulatory responses in the LP. Future research should aim to determine the impact of Cr supplementation on thermoregulatory responses in the LP as it pertains to objective measures of sweat, temperature, and exercise performance outcomes.

## Figures and Tables

**Table 1:** Demographics of the sample.

Supplement Group	Age (years)	Height (cm)	Weight (kg)	BMI (kg/m^2^)
PL (*n* = 15)	24.5 ± 4.6	167.4 ± 4.6	65.0 ± 8.2	23.1 ± 1.8
Cr (*n* = 15)	25.4 ± 7.2	164.1 ± 5.8	65.6 ± 9.2	23.6 ± 2.9

Note: Data are presented as the mean ± standard deviation. Placebo (PL): naturally menstruating (*n* = 8), hormonal contraceptive (*n* = 7); Creatine (Cr): naturally menstruating (*n* = 12), hormonal contraceptive (*n* = 3).

**Table 2:** Raw values for the body mass and fluid markers at pre- and post-supplementation visits.

	FP Pre	FP Post	LP Pre	LP Post
BM (kg)	PL: 66.14 ± 6.29 Cr: 65.52 ± 9.42	66.27 ± 6.30 65.23 ± 9.41	66.32 ± 6.19 65.21 ± 9.38	66.05 ± 6.16 65.76 ± 9.41
TBW (L)	PL: 34.80 ± 3.36 Cr: 35.27 ± 5.95	34.91 ± 3.09 35.89 ± 5.41	34.85 ± 2.87 35.22 ± 5.29	34.54 ± 2.85 36.30 ± 5.35 *
ECF (L)	PL: 13.98 ± 1.23 Cr: 14.40 ± 1.92	13.39 ± 2.71 14.49 ± 2.28	14.00 ± 0.99 14.08 ± 2.07	13.91 ± 0.98 14.36 ± 2.02 *
ICF (L)	PL: 20.85 ± 2.23 Cr: 21.65 ± 3.51	20.93 ± 2.07 21.71 ± 3.49	20.85 ± 2.01 21.14 ± 3.28	20.62 ± 1.95 21.84 ± 3.32 *

Note: Data are presented as the mean ± standard deviation. * Significance is noted from the ANCOVA with change scores covaried for hydration (USG = 1.016) and estrogen concentration (0.791 pg/mL). FP: follicular phase; LP: luteal phase; BM: body mass; TBW: total body water; ECF: extra-cellular fluid; ICF: intra-cellular fluid; PL: placebo group; Cr: creatine group.

## Data Availability

Data generated and analyzed from this study are available from the corresponding author upon reasonable request.
